# Transesplenic access in the treatment of varicose gastrointestinal bleeding. Case and technical report

**DOI:** 10.31744/einstein_journal/2020RC4934

**Published:** 2020-01-22

**Authors:** Rafael Birelo Martins, Priscila Mina Falsarella, Joaquim Maurício da Motta-Leal-Filho, Francisco Leonardo Galastri, Breno Boueri Affonso, Rodrigo Gobbo Garcia, Felipe Nasser

**Affiliations:** 1 Hospital Israelita Albert Einstein São PauloSP Brazil Hospital Israelita Albert Einstein, São Paulo, SP, Brazil.

**Keywords:** Gastrointestinal hemorrage, Esophageal and gastric varices, Embolization, therapeutic

## Abstract

Varicose gastrointestinal bleeding is one of the major causes of morbidity and mortality in patients with chronic liver disease. Endoscopic treatment is the first therapeutic line for these patients, however, for those whom this therapeutic modality fail, a broad knowledge of alternative treatment options may improve the prognosis. We describe a case of a patient who were successfully embolized from gastroesophageal varices via transsplenic access.

## INTRODUCTION

Valvular complications, including varicose bleeding, portosystemic shunts and portal vein thrombosis are major causes of morbidity and mortality in patients with crhonic liver disease.^1^ Gastric varices bleeding present death rates ranging from 25% to 55%.^2^ Currently, treatment for bleeding of varicose etiology begins with the use of vasoactive medications (terlipressin) that aim to reduce pressure regime in splanchnic circulation followed by the endoscopic treatment, when technicaly possible, in order to stop bleeding. The consensual endoscopic treatment is employed using elastic ligature, and this treatment is exclusive for thin varices (due to risk of extensive mucous injury) and cyanoacrylate injection.^3 , 4^ As an alternative, the human thrombin injection can be used with cautious due to possible thromboembolic complications.^5^

In case of endoscopy treatment failure, when the same is not technically possible or in case of early recurrence, other therapeutic options including diverse endovascular interventions such as transjugular intrahepatic portosystemic shunt (TIPS), percutaneous variceal transhepatic embolization (PVTE), partial splenic embolization (PSE), balloon-occluded retrograde transvenous obliteration (BRTO), and portal vein recanalization (in case of thrombosis).^6^

These procedures are conducted via percutatenous access, both transparietal or intravascular liver. Percutaneous trans-liver or intra-livre transjugular are the most common accesses.^2 , 7^ Other options, such as access via recanalized umbilical vein or systemic circulation via gastrorenal shunt depends on existence of alterations.^8^

However, considering the impossiblity to use these accesses, another alternative access for portal system would be the transsplenic access. This access, which use was abandoned in the beginning of 1951, due to the high associated bleeding risk, has recently returned to be employed among adult and pediatric populations, mainly after technical improvements by measures that aimed to reduce the risk of bleeding, and increase the procedure safety.^9 , 10^

## OBJECTIVE

To report a case of upper gastric varicose bleeding by embolization of left gastric vein via transsplenic access, and to discuss the technique used to access and about the safety of the procedure.

## CASE REPORT

**Figure 1:**
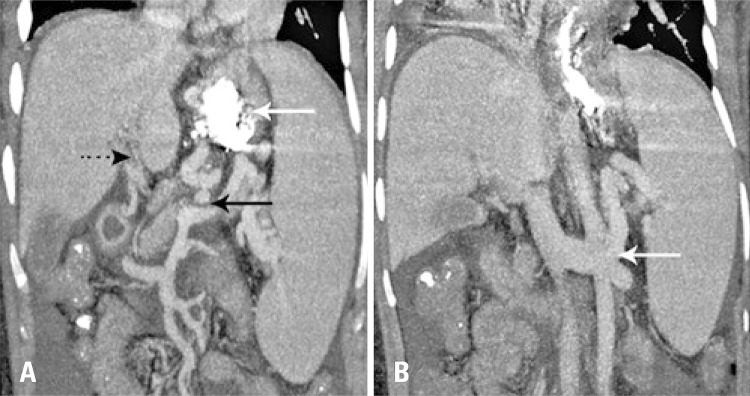
Computed tomography with intravenous contrast. (A) Enlarged left gastric vein (continous black arrow), portal thrombosis with cavernomatous transformation (pointed black arrow) and signs of previous gastresophasic varices embolization (white arrow). (B) Splenorenal shunt (white arrow)

We report a case of a 27-year-old white women with chronic thrombosis of portal vein due to portal hypertension after umbilical vein post-cateterization in neonatal period, who presented upper gastric varicose repetitive bleeding, and who had previously underwent endoscopic procedures (elastic ligature and glue injection) to treat esophagic and gastric varices. Her computed tomography (CT) identified an enlarged left gastric vein that communicated with gastroesophageal varices and splenorenal shunt ( Figura 1 ). After discussion with the multidisciplinary team, due to posterior recurrence to endoscopic treatments, a percutaneous approach was considered to stop the bleeding.

### Techique

The procedure was conducted in an hemodynamic room with patient under general anesthesia. Initially, we conducted a common right femoral artery puncture, upper mesenteric artery catheterization and an indirect portography. After confirmation of portal vein thrombosis and the presence of nutritional shunts of gastric varices, we decided to left gastric vein embolization via transsplenic access, considering that the portal vein thrombosis avoided the trans-liver access.

**Figure 2:**
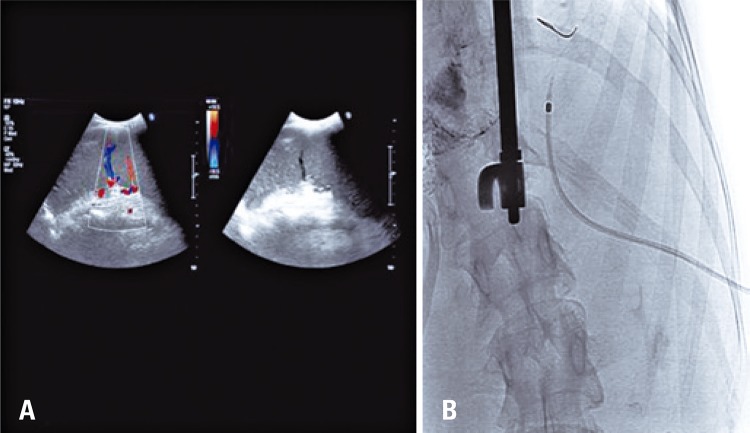
Transsplenic access. (A) Ultrasonography with Doppler of spleen to identify artery and splenic vein, and choice of the best site for puncture. (B) Transsplenic puncture. Kit of micropuncture Neff Percutaneous Assessment Set (NPAS. Cook Medical®)

The patient was placed on right oblique decubitus at 30° to improve ultrasonographic exposition of spleen, which enabled, using such position, the ecoguided puncture. The use of ultrasonography with Doppler has an important role to correct identification of vein and splenic artery ( Figure 2A ).

Puncture was conducted using 22G needle (Turner), real time ultrasound guided. A progress was made by 0.014 microguide and the implant of micropuncture kit Neff Percutaneous Assessment Set (NPAS; Cook Medical^®^) ( Figura 2B ) was carried out under fluoroscopy viewing. Venographies from the splenic vein identified splenorenal shunt of high flow. Next, superselective catheterism of proximal splenic vein identified portal vein thrombosis partially recanalized, left gastric vicariant vein and enlarged mesenteric collateral vein. Radiopac imagens in gastresophasic varices were identified using radioscopy (previous embolization with glue).

**Figure 3:**
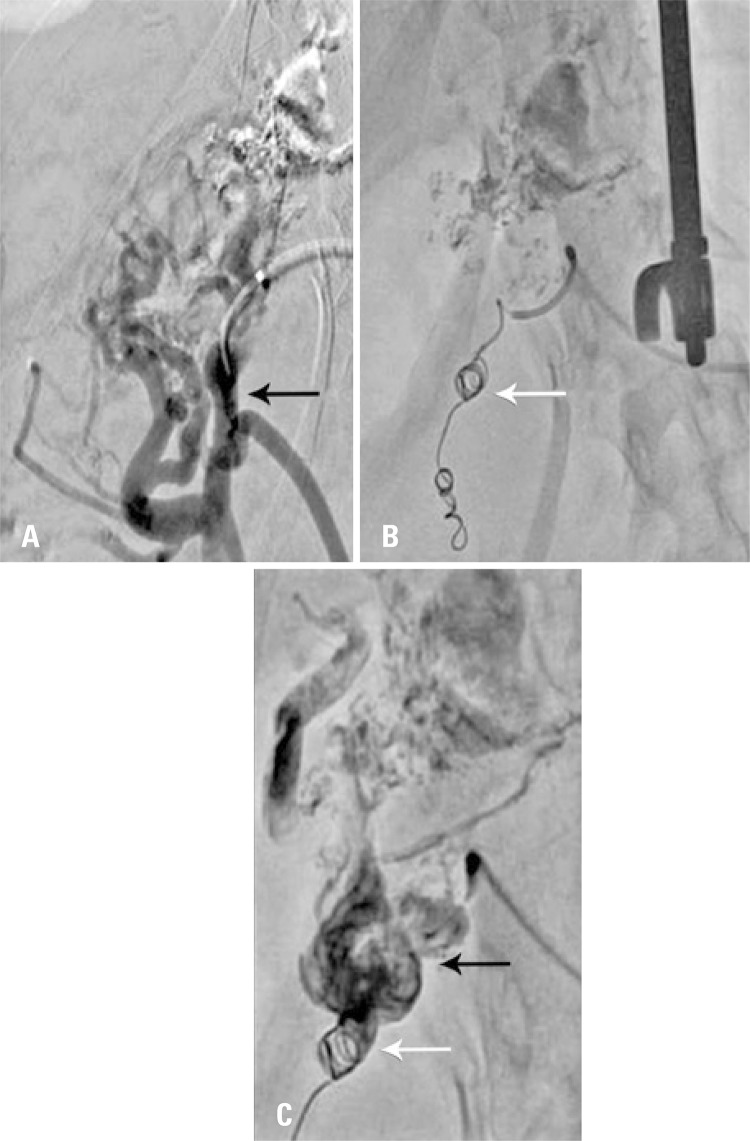
Angiography and embolization. (A) Angiography of upper mesenteric vessel branch (black arrow). (B) Embolization with fibred mole (Interlock 0.018 Boston Scientific, Marlborough, Massachusetts, USA) to avoid reflux to upper mesenteric vessel during embolizing fluid agent (white arrow). (C) Gastric left vein embolization with embolizing polimeter (Onix®) (black arrow). Lack of reflux of upper mesenteric vein due to embolizing with mole previously to polimero injection (white arrow)

Subsequentely, a superselective cateterism was conducted by dilated branch of upper mesenteric vein (UMV) that communicated with left gastric vein ( Figure 3A ). We opted by embolization for fibred mole at this time to avoid reflux for UMV during embolizing liquid agend injection ( Figure 3B ). After embolization, by superseletive cateterism of left gastric vein, we conducted gastroesophasic varices embolization with embolizing polymerous (Onix^®^; Medtronic, Minneapolis, Minnesota USA) ( Figure 3C ).

Angiographic control showed occlusion of left gastric vein, as well as preserved flow preserved in splenic vein. In the end of the procedure, the traject via parenchima splenic was embolized with Gelfoam^®^ torped, previous to total removal of the i introducing instrument, to reduce bleeding risks.

**Figure 4:**
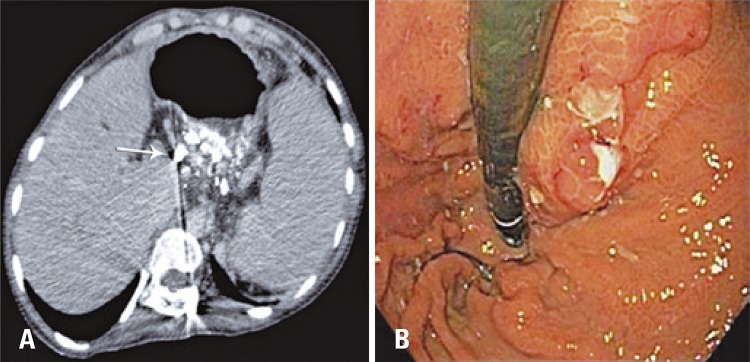
Control exam after 30 days. (AG) Computed tomography after 30 days showing gastric varices filled with fluid agent (Onix®), white arrow. (B) Upper digestive endoscopy of control 30 days after the procedure

In post-operatory follow-up, the patient conduted a control CT and new upper digestive endoscopy, which showed the presence of embolizing agent and reduction of variceal size, without evidence of recent bleeding ( Figure 4 ). The follow-up was maintained for 4 years, without new bleeding episodes.

## DISCUSSION

In this case report, lack of endoscopy treatment and impossiblity to trans-liver or transjugular access due to presence of chronic thrombosis of portal vein were the main factors to opt for the transsplenic access.

The transsplenic access is used as a less invasive alternative than surgery, and this access has been employed for treatment of chronic tromboses of portal vein, such as via access for portal vein embolization pre-hepactectomy and varices embolization.^11^

This tecnique evolution as well as possibility of use with hemostatic agent for embolization of the traject, enabled more safety with less complications, and it has been adopted for adult and pediatric population. The use of Gelfoam^®^ is safe according to current studies published in the literature, but other alternatives exist, such as use of Amplatzer^®^ plug.^11 , 12^

A Korean study including 26 patients showed to be factible the transsplenic access and reported techincal success in 24 of 27 procedures (26 patients) performed. Unsuccessful cases occurred due to dissection of splenic vein, tortuosity of splenic vein and possibility of trans-live access according to the procedure due to portal recanalization.^13^

To this access, there is a need to use ultrasound, for adequate identification of artery and splenic vein, as well as to guide puncture, aiming to reduce risk of inadverted puncture of non-target structures. Puncture must be done with micropuncture kit to reduce bleeding risk. In specific situations, proximal embolization of proximal vascular segment can be done, aiming to reduce the possibility of distal embolization, when posteriorly the fluid agent or particulate agent is used. In this patient, the use of moles avoided the progression of fluid agent (Onix^®^) for non-target vessel (in case of UMV), which provided a safer and precision to the embolization of the segment to be treated. Considering the portal vein thrombosis with cavernomatous transformation, time of occlusion (since the neonatal period), and the risk of collateral ocllusion, the recanalization of portal vein was not carried out.

## CONCLUSION

In our case, transsplenic access was considered safe. When this procedure is conducted by a trained team by employing adequate materials and techniques, this can be useful for patients who present contraindication or impossibility to other types of access. The endovascular treatment using percutaneous micropuncture technique enabled a safe and effective embolization, and complications in the follow-up were observed. Procedures conducted using transsplenic access are safe and present low rate of complications, however, further and more robust studies are warranted to analyze this procedure safety.
