# Supplementary material for: Transesplenic access in the treatment of varicose gastrointestinal bleeding. Case and technical report
Source: Einstein (Sao Paulo). 2020 Jan 22;18:eRC4934. doi: 10.31744/einstein_journal/2020RC4934 (PMC6980296; doi:10.31744/einstein_journal/2020RC4934)
Supplement: Translation

# Translation: Uso do acesso transesplênico no tratamento do sangramento gastrointestinal varicoso. Relato de caso e técnica

**Authors:** Rafael Birelo Martins, Priscila Mina Falsarella, Joaquim Maurício da Motta-Leal-Filho, Francisco Leonardo Galastri, Breno Boueri Affonso, Rodrigo Gobbo Garcia, Felipe Nasser

**Affiliations:** 1 Brasil Hospital Israelita Albert Einstein, São Paulo, SP, Brasil.

**Keywords:** Hemorragia gastrointestinal, Varizes esofágicas e gástricas, Embolização terapêutica

## Abstract

O sangramento gastrointestinal varicoso está entre as maiores causas de morbimortalidade nos paciente com doença hepática crônica. O tratamento endoscópico é a primeira linha terapêutica neste pacientes, porém naqueles que apresentam falha nesta modalidade terapêutica, o amplo conhecimento de opções alternativas de tratamento pode melhorar o prognóstico. Descrevemos um caso de paciente submetido à embolização com sucesso de varizes gastresofágicas por acesso transesplênico.

## INTRODUÇÃO

Complicações vasculares, incluindo sangramento varicoso, *shunts* portossistêmicos e trombose da veia porta, são as maiores causas de morbidade e mortalidade em pacientes com doença hepática crônica.^1^ O sangramento por varizes gástricas possui taxas de mortalidade na ordem de 25 a 55%.^2^ Atualmente, o tratamento do sangramento de etiologia varicosa é iniciado com uso de medicações vasoativas (terlipressina), visando à redução do regime pressórico na circulação esplâncnica, acompanhado de tratamento endoscópico, quando tecnicamente possível, com a finalidade de cessar o sangramento. O tratamento endoscópico consensualmente empregado é realizado com ligadura elástica, exclusivo para varizes de fino calibre (devido ao risco de lesão extensa da mucosa) e injeção de cianoacrilato.^3 , 4^ Como alternativa, pode ser utilizada a injeção de trombina humana de forma mais cautelosa, devido à possibilidade de complicações tromboembólicas.^5^

No caso de falha do tratamento endoscópico, quando o mesmo não é tecnicamente possível ou nos casos de recidiva precoce, outras opções terapêuticas incluem intervenções endovasculares diversas, como comunicação portossistêmica intra-hepática via transjugular (TIPS - *transjugular intrahepatic portosystemic shunt* ), embolização varicosa trans-hepática percutânea (PVTE - *percutaneous variceal transhepatic embolisation* ), embolização esplênica parcial (PSE - *partial splenic embolisation* ) embolização trans--hepática, obliteração transvenosa retrógrada com balão (BRTO - *balloon-occluded retrograde transvenous obliteration* ) e recanalização da veia porta (nos casos em que há trombose).^6^ Esses procedimentos são realizados por acesso percutâneo, seja transparietal hepático ou intravascular. Os acessos trans-hepático percutâneo ou transjugular intra-hepático são os mais comuns.^2 , 7^ Outras opções, como o acesso pela veia umbilical recanalizada ou pela circulação sistêmica via *shunt* gastrorrenal, dependem da existência dessas alterações.^8^

No entanto, frente à impossibilidade de utilizar essas vias, uma outra alternativa de acesso ao sistema portal seria o transesplênico. Este acesso, abandonado no início de seu uso em 1951, devido ao elevado risco de sangramento associado, recentemente voltou a ser utilizado nas populações adulta e pediátrica, após melhora da técnica, com medidas que visaram reduzir o risco de sangramento, aumentando sua segurança.^9 , 10^

## OBJETIVO

Relatar um caso de hemorragia digestiva alta varicosa tratado por embolização da veia gástrica esquerda com acesso transesplênico, além de discutir a técnica utilizada para o acesso e a segurança do procedimento.

## RELATO DE CASO

Paciente do sexo feminino, branca, 27 anos, com trombose crônica da veia porta secundária à hipertensão portal pós-cateterização da veia umbilical no período neonatal, apresentando quadros de hemorragia digestiva alta varicosa de repetição, previamente submetida a procedimentos endoscópicos (ligadura elástica e injeção de cola) para tratamento de varizes esofágicas e gástricas. Realizada tomografia computadorizada (TC), que identificou veia gástrica esquerda dilatada, com comunicação com varizes gastresofágicas e *shunt* esplenorrenal ( Figura 1 ). Após discussão multidisciplinar, devido à recidiva posterior a tratamentos endoscópicos, optou-se pela abordagem percutânea do sangramento.

**Figura 1:** Tomografia computadorizada com contraste endovenoso. (A) Veia gástrica esquerda dilatada (seta preta contínua), trombose de porta com recanalização cavernomatosa (seta preta pontilhada) e sinais de embolização prévia de varizes gastresofágicas (seta branca). (B) *Shunt* esplenorrenal (seta branca)

### Técnica

O procedimento foi realizado em sala de hemodinâmica sob anestesia geral. Inicialmente, foram realizadas punção da artéria femoral comum direita, cateterização da artéria mesentérica superior e portografia indireta. Após confirmação de trombose portal e presença de *shunts* nutridores das varizes gástricas, optou-se por embolização da veia gástrica esquerda por acesso transesplênico, já que a trombose portal impediu o acesso trans-hepático.

O paciente foi posicionado em decúbito oblíquo direito a 30°, para melhor exposição ultrassonográfica do baço, facilitando, desta forma, a punção ecoguiada. O uso da ultrassonografia com Doppler tem papel fundamental para correta identificação de veia e artéria esplênica ( Figura 2A ).

**Figura 2:** Acesso transesplênico. (A) Ultrassonografia com Doppler do baço para identificação da artéria e da veia esplênica, e escolha do melhor sítio de punção. (B) Punção transesplênica. *Kit* de micropunção *Neff Percutaneous Assessment Set* (NPAS; Cook Medical®)

A punção foi realizada com agulha 22G (Turner), guiada em tempo real por ultrassonografia. Progrediu-se com microguia 0,014” e foi feito implante de *kit* de micropunção *Neff Percutaneous Assessment Set* (NPAS; Cook Medical^®^) ( Figura 2B ) sob visão fluoroscópica. As venografias a partir da veia esplênica identificaram *shunt* esplenorrenal de alto fluxo. Em seguida, o cateterismo superseletivo da veia esplênica proximal identificou trombose portal parcialmente recanalizada, veia gástrica esquerda vicariante e veia colateral mesentérica dilatada. Imagens radiopacas em varizes gastresofágicas foram identificadas à radioscopia (embolização prévia com cola).

Na sequência, foi realizado o cateterismo superseletivo do ramo dilatado da veia mesentérica superior (VMS), que se comunicava com a veia gástrica esquerda ( Figura 3A ). Optou-se por embolização com mola fibrada neste momento, para evitar refluxo para a VMS durante injeção do agente líquido embolizante ( Figura 3B ). Após a embolização, por meio de cateterismo superseletivo da veia gástrica esquerda, foi realizada embolização das varizes gastresofágicas com polímero embolizante (Onix^®^; Medtronic, Minneapolis, Minnesota EUA) ( Figura 3C ).

**Figura 3:** Angiografia e embolização. (A) Angiografia de ramo da veia mesentérica superior (seta preta). (B) Embolização com mola fibrada (Interlock 0,018, Boston Scientific, Marlborough, Massachusetts, EUA) para evitar refluxo para a veia mesentérica superior durante injeção do agente líquido embolizante (seta branca). (C) Embolização da veia gástrica esquerda com polímero embolizante (Onix®) (seta preta). Ausência de refluxo para veia mesentérica superior, devido à embolização com mola previamente à injeção do polímero (seta branca)

O controle angiográfico demonstrou oclusão da veia gástrica esquerda, bem como fluxo preservado em veia esplênica. Ao final do procedimento, o trajeto através do parênquima esplênico foi embolizado com torpedo de Gelfoam^®^, prévio à retirada completa do introdutor, para minimizar riscos de sangramento.

No seguimento pós-operatório, a paciente realizou TC de controle e nova endoscopia digestiva alta, evidenciando presença do agente embolizante e redução no calibre das varizes, sem evidências de sangramento recente ( Figura 4 ). Manteve seguimento por 4 anos, sem novos episódios de sangramento.

**Figura 4:** Exames de controle após 30 dias. (A) Tomografia computadorizada após 30 dias mostra varizes gástricas preenchidas pelo agente líquido (Onix®), seta branca. (B) Endoscopia digestiva alta de controle após 30 dias do procedimento

## DISCUSSÃO

Neste caso, a falha do tratamento endoscópico e a impossibilidade do acesso trans-hepático ou transjugular devido à presença de trombose crônica da veia porta foram os principais fatores para a escolha do acesso transesplênico.

O acesso transesplênico é utilizado como alternativa menos invasiva que a cirurgia, sendo empregado para tratamento de trombose crônica da veia porta, como via de acesso para embolizaçãoo de veia porta pré-hepatectomia e na embolizaçãoo de varizes.^11^

A evolução da técnica, bem como a possibilidade do uso de agente hemostático para embolização do trajeto, trouxe mais segurança com menos complicações, sendo usado na população adulta e pediátrica. O uso do Gelfoam^®^se mostra seguro segundo a literatura atual, existindo outras alternativas, como, por exemplo, o uso do *plug* de Amplatzer^®^.^11 , 12^

Estudo coreano com 26 pacientes mostrou ser factível o acesso transesplênico, com sucesso técnico em 24 de 27 procedimentos (26 pacientes) selecionados. Os casos de insucesso se deram por dissecção da veia esplênica, tortuosidade da veia esplênica e possibilidade de acesso trans-hepático no segundo procedimento, devido à recanalização portal.^13^

Para esse acesso, faz-se necessário o uso do ultrassom, para adequada identificação da artéria e veia esplênica, bem como para guiar a punção, visando reduzir o risco de punção inadvertida de estruturas não alvo. A punção deve ser realizada com *kit* de micropunção, para minimizar o risco de sangramento. Em situações específicas, a embolização proximal de um segmento vascular pode ser realizada, visando minimizar a possibilidade de embolização distal, quando posteriormente é utilizado agente líquido ou agente particulado. Neste paciente, o uso de molas evitou a progressão do agente líquido (Onix^®^) para um vaso não alvo (no caso a VMS), proporcionando mais segurança e precisão na embolização do segmento a ser tratado. Considerando a trombose portal com transformação cavernomatosa, o tempo de oclusão (desde o período neonatal) e o risco de oclusão de colaterais, a recanalização da veia porta não foi realizada.

## CONCLUSÃO

O acesso transesplênico mostrou-se seguro neste caso. Quando realizado por equipe treinada, empregando materiais e técnicas adequados, pode ser útil naqueles pacientes que apresentam contraindicação ou impossibilidade de outros acessos. Neste caso, o tratamento endovascular com técnica de micropunção percutânea transesplênica permitiu embolização segura e efetiva, sem complicações no seguimento. Procedimentos realizados por essa via são seguros e têm baixa taxa de complicações, porém estudos maiores e mais robustos são necessários para analisar sua segurança.
